# Does thiol/disulfide homeostasis affect the number of metaphase 2 oocyte in the treatment of *in vitro* fertilization?

**DOI:** 10.2144/fsoa-2019-0132

**Published:** 2020-01-14

**Authors:** Ayse Z Ozdemir, Pervin Karlı, Salim Neşeli̇oğlu

**Affiliations:** 1 Ondokuz Mayıs University IVF Center, Ondokuz Mayıs University Hospital, Samsun, Turkey; 2 Amasya University, Department of Obstetrics & Gynecology, Amasya, Turkey; 3 Yıldırım Beyazıd University Department of Biochemisrty, Ankara, Turkey

**Keywords:** antioxidants, embryo, *in vitro* fertilization success, infertility, metaphase 2, oocyte maturation, oxidative stress, pregnancy, serum, thiol/disulfide homeostasis

## Abstract

**Aim:**

To investigate the effect of thiol/disulfide homeostasis on the number of oocytes retrieved and metaphase 2 (M2) oocytes in patients undergoing *in vitro* fertilization.

**Method:**

A prospective study of 94 patients who were admitted to the *in vitro* fertilization clinic was conducted. Serum samples were taken on the oocyte pick-up day and kept until the analysis. Thiol and disulfide were measured in order to evaluate the total thiol/disulfide.

**Results:**

A statistically significant correlation was observed between disulfide and M2.

**Conclusion:**

This study provides an inexpensive and noninvasive method to measure oxidative stress, and suggests that there is a positive correlation between the number of M2 and disulfide, resulting in low-impact oxidative stress.

## Lay abstract

*In vitro* fertilization success is a most important issue, and a lot of factors have been shown to affect its success. Oxidative stress is one of the most important of these factors. There are a lot of ways to measure oxidative stress, and here we wanted to show the effect of oxidation in *in vitro* fertilization success by using a new, cheaper method. The study provides an important contribution to the literature by showing the positive effect of a small amount of oxidative stress on oocyte maturation.

In assisted reproductive technology cycles, take home baby rate has not yet reached the desired level worldwide because of several unknown factors. However, many factors such as endometrial thickness, trilaminar appearance of the endometrium, traumatization during embryo transfer and oxidative stress (OS) affect the success of *in vitro* fertilization (IVF) as stated in several studies [1,2].

While certain levels of oxygen radicals are required for normal reproduction, increasing levels of the oxygen radicals damage reproductive cells [3]. Increased free oxygen radicals disrupt nearly all components of the cell – that is, protein, lipids, nucleic acids and carbohydrate structures and so on [4]. By doing so, it damages the mitochondrial function and therefore reduces energy production. This situation negatively affects oocyte maturation, which is an energy-dependent process, and therefore oocyte fertilization.

Thiols are of the class of organic compounds known as mercaptans, and their forms containing sulfhydryl groups have a critical role in any OS situation in cells [5]. They enter the oxidative reaction through oxidants and form disulfide bonds [6].

The thiol/disulfide ratio has a critical function in antioxidant protection, detoxification, signal transduction, enzymatic activity regulation, apoptosis and mechanisms of cellular signaling [7]. Moreover, understanding the dynamics of thiol/disulfide homeostasis is essential because its abnormality provides information about the pathogenesis of various diseases [8,9].

In demonstrating OS, disulfide bridges can be considered as markers. When cysteine residues in OS increase, they stimulate the development of a reversible reaction between low-molecular-mass thiols and protein thiol groups. Thiol/disulfide homeostasis is achieved by the reduction of disulfide bonds to thiol groups [10]. Measuring the thiol values only provides information about the antioxidant system. Generally, the level of plasma thiol is measured by the classical Ellman reagent, 5,5 5-dithiobis-(2-nitrobenzoic) acid. However, a new test method has been developed by Erel and Neşelioğlu that defined the homeostasis of thiol/disulfide and allowed the evaluation of the thiol/disulfide status. Erel *et al.* revealed that the thiols were the most abundant antioxidant components in the serum [11]. Evaluating the thiol/disulfide homeostasis, which is the dynamic redox system of organisms, using the method developed by Erel *et al.* would provide more objective data.

Therefore, our study will provide an indirect, noninvasive and inexpensive measurement of OS, which may have a negative effect on an embryo. As IVF is an expensive and troublesome method, our new method may contribute positively to IVF success and guide new antioxidant treatment methods.

## Materials & methods

This study was approved by the Ondokuz Mayıs University Ethics Committee (OMU KAEK 2018-14) All patients included in this study were admitted between July and October 2018 at the Center for *In-vitro* Fertilization of Ondokuz Mayıs University Hospital, All patients signed an informed consent form.

### Patient selection

Patients those with no detected sperm from male partner through testicular sperm extraction were excluded from the study.

Patients were also excluded based on age (older than 42 years), any medications used, hepatic or renal diseases, being a smoker, having systemic infections or endometriosis. In addition, patients with oligo/anovulation, polycystic ovary appearance on the ultrasonography, increased luteinizing hormone/follicle stimulating hormone (LH/FSH) ratio, hirsutism clinical or laboratory findings, and hyperprolactinemia or thyroid hormone disorder were excluded from the study.

In selecting patients, attention was paid to ensure that they were diagnosed with male factor, unexplained infertility or diminished ovarian reserve (DOR). We classified patients in three groups (unexplained infertility = Group 1, DOR = Group 2; and male factor = Group 3).

For the male factor diagnosis, we included patients who were below the specified sperm parameters based on the results of two semen analyses that were performed after 48–72 h of sexual abstinence in compliance with the WHO criteria [12]. Male factor infertility is diagnosed as the presence of ≥1 abnormalities in the semen analysis or the presence of inadequate sexual or ejaculatory function.

For the diagnosis of unexplained infertility, following the ESHRE Unexplained Infertility Workshop, we included patients for whom the cause of infertility was still undetermined or the infertility reasons cannot be explained through semen analysis, evaluation of ovulation and evaluation of tubal patency [13,14].

For the poor ovarian reserve diagnosis, we included patients fulfilling the Bologna criteria, that is, at least two of the following three features must be present: advanced maternal age (≥40 years) or any other risk factor for POR; a previous POR (≤3 oocytes with a conventional stimulation protocol); and an abnormal ovarian reserve test (i.e., AFC, 5–7 follicles or AMH, 0.5–1.1 ng/ml) [14].

### Ovulation induction

The second or third day of menstruation ovarian stimulation was performed using recombinant gonadotropins (Gonal F, Merck-Serono, Rome, Italy). The dose of gonadotropins was adjusted ultrasonographically to the ovarian response follow-up. As soon as the dominant follicle diameter reached 12–14 mm, the daily gonadotropin-releasing hormone (GnRH) antagonist (cetrotide, cetrorelix acetate, Merk Serono, UK) was given until at least three follicles reached up to 18 mm in diameter (until HCG injection day). The OPU was performed 34–36 h after the HCG injection, then the intracytoplasmic sperm injection (ICSI) was performed. The embryo transfer was performed on the third day of OPU.

Each of the patients was given 100 mg progesterone intramuscular (progestan 50 mg, Kocak) and 6 mg estradiol orally (estrofem 2 mg, Novo Nordisk) as luteal support following the day of OPU.

The following data were recorded for all patients: age; infertility duration; FSH and estradiol (E2) tested on the second or third day of the menstrual cycle (D2–D3); endometrial thickness, estradiol, progesterone and LH tested on the HCG day; daily and mean gonadotropin dose used; mean number of days on which the patient was given gonadotropin;

The primary outcome is pregnancy, as confirmed by positive HCG test 14 days after embryo transfer.

The secondary outcomes were mean number of retrieved oocytes, metaphase 2 (M2) oocytes formed, number of embryos formed and pronucleus (PN) Number.

### Sample collection

The serum samples were taken from the patients on the day of OPU. Then, these samples were separated after centrifugation at 100 g for 10 min and stored at -80°C until analysis.

### Serum thiol/disulfide homeostasis parameter measurements

The tests of thiol/disulfide homeostasis were carried out using the automated spectrophotometric method defined by Erel and Neşelioğlu [11]. In brief, disulfide bonds were lowered to free function thiol groups with sodium borohydride. The unused reductant sodium borohydride was consumed and removed with formaldehyde to prevent the reduction of DTNB (5, 5′-dithiobis-[-nitrobenzoic]-acid). Then, all of the thiol groups including the reduced and native thiol groups were determined after the reaction with DTNB. The dynamic disulfide amount was determined to be half of the difference between the total thiols and native thiols. After determining the native and total thiols, we calculated the disulfide amounts, disulfide/total thiol percent ratio (SS/SH + SS), disulfide/native thiol percent ratio (SS/SH), and native thiol/total thiol percent ratios (SH/SH + SS). The results of these calculations are provided as indexes 1, 2 and 3 (Table 2).

**Table 1.** Comparison results of some measurements according to the groups.

	Group 1 (n = 26)				Group 2 (n = 16)				Group 3 (n = 20)				p^†^
	Mean ± SD	Med.	Min.	Max.	Mean ± SD	Med.	Min.	Max.	Mean ± SD	Med.	Min.	Max.	
Patients’ age	31.38 ± 5.13^‡^	30.50	22.00	39.00	36.75 ± 4.80^‡^	40.00	23.00	42.00	29.84 ± 4.77^‡^	30.00	20.00	39.00	<0.001
Duration of infertility	7.06 ± 5.07	6.00	1.00	20.00	6.15 ± 5.84	5.00	1.00	23.00	6.24 ± 4.57	5.00	1.00	16.00	0.464
FSH	8.18 ± 4.45^‡^	6.50	2.90	24.00	13.00 ± 6.42^‡^	12.10	3.00	33.00	7.33 ± 3.05^‡^	7.00	3.60	18.00	<0.001
Day 2–3 estradiol	42.59 ± 20.47	42.00	5.00	89.00	38.40 ± 36.79	29.50	5.00	188.00	41.52 ± 21.73	39.00	5.00	83.00	0.085
hCG day estradiol	1369.78 ± 690.9^‡^	1280.0	180.00	3022.0	524.63 ± 441.5^‡^	316.00	68.00	1678.00	1477.16 ± 1014.9^‡^	1193.00	170.00	4365.00	<0.001
hCG day Progesterone	0.430 ± 0.330^‡^	0.30	0.10	1.40	.510 ± 1.310^‡^	0.20	0.05	8.40	0.530 ± 0.310^‡^	0.50	0.10	1.40	0.002
hCG day LH	2.71 ± 2.97^‡^	1.50	0.40	13.00	5.70 ± 4.81^‡^	4.40	0.10	22.00	1.78 ± 1.37^‡^	1.60	0.10	5.20	<0.001
Gonadotropine dose used	370.59 ± 96.62	337.5	225.00	600.0	381.88 ± 114.91	387.50	150.00	600.00	357.00 ± 104.46	300.00	225.00	600.00	0.421
Duration of gonadotropin used	9.71 ± 2.39	10.00	6.00	15.00	8.90 ± 2.73	9.00	3.00	16.00	9.04 ± 1.70	9.00	6.00	12.00	0.431
Endometrial thickness	8.47 ± 1.81^‡^	8.00	6.00	13.00	7.88 ± 1.28^‡^	8.00	5.00	11.00	9.04 ± 1.62^‡^	9.00	6.00	12.00	0.025
Number of collected oocytes	7.97 ± 4.32^‡^	8.00	1.00	18.00	2.08 ± 2.19^‡^	1.00	1.00	8.00	8.88 ± 6.08^‡^	8.00	3.00	27.00	<0.001
M2 oocyte	6.94 ± 4.10^‡^	6.50	1.00	16.00	2.52 ± 1.86^‡^	2.00	0.00	8.00	7.32 ± 4.31^‡^	7.00	3.00	20.00	<0.001
Number of Embryos	5.57 ± 3.57^‡^	6.00	0.00	13.00	2.06 ± 1.20^‡^	2.00	0.00	4.00	4.71 ± 3.65^‡^	4.00	0.00	13.00	0.002
PN	5.71 ± 3.59^‡^	6.00	0.00	13.00	2.41 ± 1.33^‡^	2.00	0.00	5.00	5.19 ± 3.64^‡^	4.00	2.00	15.00	0.002

† Kruskal-Wallis Test; values given in the table are mean ± SD.

‡ Indicate the difference between the indication types (*post-hoc* test with Bonferroni correction).

FSH: Follicle stimulating hormone; LH: Luteinizing hormone; M2: Metaphase 2; Max.: Maximum, Med.: Medium; Min.: Minute; PN: Pronucleus; SD: Standard deviation.

**Table 2.** Comparison results of thiol measurements according to the groups.

	Group 1 (n = 26)				Group 2 (n = 16)				Group 3 (n = 20)				p^†^
	Mean ± SD	Med.	Min.	Max.	Mean ± SD	Med.	Min.	Max.	Mean ± SD	Med.	Min.	Max.	
Albumin	4.77 ± 0.28	4.80	3.85	5.41	4.85 ± 0.26	4.84	3.98	5.43	4.74 ± 0.16	4.71	4.41	5.04	0.078
IMA	0.750 ± 0.100	0.77	.049	0.94	0.750 ± 0.090	0.75	0.58	0.95	0.750 ± 0.070	0.77	0.52	0.85	0.844
Native thiol	262.28 ± 68.60	276.05	55.58	343.26	277.07 ± 50.18	286.03	129.69	359.41	265.61 ± 68.51	276.46	20.17	362.50	0.716
Total thiol	300.18 ± 59.28	306.05	150.58	391.26	307.60 ± 53.20	311.10	147.69	399.41	302.97 ± 63.44	311.41	76.17	394.50	0.903
Disulfide	18.95 ± 9.96	16.00	6.00	52.00	15.26 ± 3.71	15.00	8.00	26.00	18.68 ± 7.71	16.00	11.00	43.00	0.142
Index 1	10.24 ± 15.91	5.43	3.18	85.46	5.64 ± 1.64	5.36	3.83	12.01	12.35 ± 26.70	5.46	4.41	138.80	0.270
Index 2	6.99 ± 6.04	4.90	2.99	31.54	5.03 ± 1.25	4.84	3.56	9.68	7.22 ± 6.78	4.92	4.06	36.76	0.270
Index 3	86.02 ± 12.08	90.20	36.91	94.02	89.94 ± 2.51	90.31	80.63	92.88	85.57 ± 13.56	90.16	26.48	91.89	0.270

† Kruskal–Wallis Test result; values given in the table are average ± SD.

IMA: Ischemia-modified albumin; Max.: Maximum, Med.: Medium; Min.: Minute; SD: Standard deviation.

### Statistical analysis

For calculating the sample width (magnitude), power was determined by taking at least 0.80 and type 1 error 0.05 (NCSS PASS [ver.11]). For the power analysis test, the minimum number of subjects requested in each group was 13 in order to observe a significant difference between the group average of 1.50 units. Descriptive statistics for continuous variables were median, mean, standard deviation, min–max. The Shapiro–Wilk test (n < 50) was used to identify whether the continuous variables in the study were distributed normally. It was determined that the variables were not normally distributed; hence, nonparametric tests were conducted. Furthermore, the Mann–Whitney U test was applied to compare the measurements in proportion to the gestational status. Continuous variables were compared among the three groups using Kruskal–Wallis test. Then, the Spearman correlation coefficient was calculated to identify the relationship between the continuous variables. For the calculations, the statistical significance level (α) was 5% and SPSS (IBM SPSS for Windows, ver.24) statistical package program was used.

## Results

### Assay details including sensitivity, specificity & limits

The mean percent recovery was 98–101%.

The upper limit of the linearity for the native thiol measurement was 4000 μM. Linearity of the total thiol measurement was also dependent on the amounts of NaBH_4_ and formaldehyde concentrations. The upper limit of the linearity for the disulphide measurement was 2000 μM.

The lower detection limit, defined as the mean value of zero calibrator + 3 standard deviations (SDs), was 2.8 μM.

Analytical sensitivity was found to be 7.9 × 10−4 absorbance/amount, [A × (μM)−1].

It was found that hemoglobin, EDTA, citrate and oxalate did not interfere with the assay development, but bilirubin did negatively interfere with the assay. Lipaemic and uraemic plasma samples did not interfere with the assay. Plasma and serum samples can be used as samples [11].

**Figure 1.**
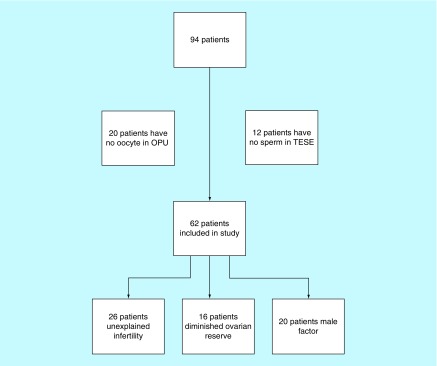
Patient demographics. A total of 94 patients who underwent *in vitro* fertilization/intracytoplasmic sperm injection treatment were included in the study. OPU: Oocyte pick-up; TESE: Testicular sperm extraction.

The study was continued with a total of 62 patients; 26 of whom were diagnosed with unexplained infertility, 16 with DOR, and 20 with male factor (Figure 1).

The comparison of results of some measurements according to groups are shown in Table 1. A statistically significant difference was observed between groups (p < 0.05) with regard to the patient age and FSH tested on the second day of menstruation, and estradiol, progesterone, LH tested on the HCG day, endometrial thickness and number of retrieved oocytes, M2s, number of embryos, and PN. Differently formed groups are denoted by different lowercase letters (a, b and c). By contrast, no statistically significant difference was observed between the groups and other variables except for these measurements (p > 0.05). The comparison of results of albumin, IMA, and thiol measurements (native thiol, total thiol, disulfide and indexes 1, 2 and 3) according to the groups are given in Table 2. There was no statistically significant difference between groups for all thiol measurements (p > 0.05). Comparing the results of albumin, IMA and thiol measurements according to pregnancy status, there was no statistically significant difference (Table 3). For the correlation relationship between the number of retrieved oocytes, PN and M2 and the measurements of albumin, IMA and thiol, a statistically significant relationship between ‘disulfide’ and ‘M2’ (p < 0.05) was observed. This relationship was positive with 22.6%. The result implies that as ‘disulfide’ increases, ‘M2’ also increases. There was no statistically significant relationship between other variables/measurements (Table 4).

**Table 3.** Comparison results of thiol measurements according to pregnancy status.

	Not pregnant (40)					Pregnant (n = 22)					p^†^
	Mean	SD	Med.	Min.	Max.	Mean	SD	Med.	Min.	Max.	
Albumin	4.79	0.26	4.79	3.85	5.43	4.76	0.29	4.79	4.26	5.30	0.751
IMA	0.75	0.09	0.77	0.49	0.92	0.75	0.10	0.74	0.58	0.94	0.993
Native thiol	261.56	69.81	279.60	20.17	343.26	276.96	59.77	307.03	160.77	362.50	0.503
Total thiol	297.49	61.61	311.10	76.17	391.26	313.57	58.96	349.22	200.56	394.50	0.470
Disulfide	17.97	8.91	15.00	8.00	52.00	18.31	8.52	16.00	6.00	42.00	0.395
Index 1	11.54	23.00	5.38	3.83	138.80	7.22	5.82	5.49	3.18	26.12	0.778
Index 2	7.01	6.74	4.85	3.56	36.76	5.98	3.51	4.95	2.99	17.16	0.778
Index 3	85.98	13.49	90.29	26.48	92.88	88.04	7.02	90.10	65.68	94.02	0.778

† Mann–Whitney U test.

IMA: Ischemia-modified albumin; Max.: Maximum, Med.: Medium; Min.: Minute; SD: Standard deviation.

**Table 4.** Spearman correlations.

		Collected oocyte number	PN	M2
Albumin	r	-0.136	-0.189	-0.183
	p	0.187	0.135	0.098
	N	96	64	83
IMA	r	-0.096	-0.185	-0.163
	p	0.351	0.143	0.142
	N	96	64	83
Native thiol	r	-0.018	0.059	-0.068
	p	0.862	0.644	0.539
	N	96	64	83
Total thiol	r	0.030	0.096	-0.011
	p	0.770	0.450	0.923
	N	96	64	83
Disulfide	r	0.189	0.109	0.226^†^
	p	0.065	0.389	0.040
	N	96	64	83
Index 1	r	0.070	-0.063	0.077
	p	0.501	0.620	0.491
	N	96	64	83
Index 2	r	0.110	-0.016	0.137
	p	0.287	0.898	0.217
	N	96	64	83
Index 3	r	-0.110	0.016	-0.137
	p	0.287	0.898	0.217
	N	96	64	83

† p < 0.05; r: Spearman's correlation coefficients.

IMA: Ischemia-modified albumin; M2: Metaphase 2; PN: Pronucleus.

## Discussion

In our study thiol/disulfide homeostasis did not differ significantly. However, a positive correlation occurs between isolated disulfide levels and M2 oocytes, which has proven that a small amount of OS is necessary for oocyte maturation.

Recent studies have also emphasized that OS has important effects in female infertility [15]. OS indicates increased reactive oxygen species (ROS) due to reduced antioxidant capacity [16]. The impaired antioxidant–oxidant balance leads to changes in cells and macromolecules and, eventually, cell death and dysfunction [17]. OS, oocyte maturation, ovarian steroidogenesis and corpus luteum formation play a role in nearly all physiological functions of the reproductive system such as fertilization, embryo development and pregnancy [18]. Only one aspect of this balance was measured using the method developed by Ellman in 1979. However, the information about the directions of thiol/disulfide balance was obtained using the method created by Erel and Neşelioğlu [11].

ROS and the free and non-free radical oxygenated molecules are produced in many physiological processes and are beneficial in low concentrations [19]. Thiol/disulfide homeostasis did not differ significantly in our study. However, a positive correlation occurs between isolated disulfide levels and M2 oocytes, which has proven that a small amount of OS is necessary for biological systems, even at low levels. The higher-than-normal OS in cells causes damage in proteins, DNA and lipids. Organisms regulate ROS with antioxidant mechanisms [20]. Furthermore, ROS that are produced in follicles play a role in folliculogenesis and ovulation [21]. In female infertility, OS plays an etiopathogenic role. In recent studies, it has been suggested that OS increases in diseases such as polycystic ovary syndrome (PCOS), and the correction of this oxidative environment may be beneficial in infertility treatment [22].

Thiol/disulfide levels were measured in patients with premature ovarian failure. The levels of disulfide were observed to increase, and a positive correlation was found between the FSH levels and this increase. Moreover, the results of this study suggested that antioxidant support may be beneficial for women with a family history of premature ovarian failure [23]. Tola *et al.* studied follicular fluid thiol/disulfide homeostasis in PCOS and observed that the homeostasis deteriorated. With this homeostasis disruption, a positive correlation with fertilization rates in the IVF process was found in PCOS patients. In order to increase the success of IVF in PCOS patients, thiol/disulfide homeostasis needs to be improved [24].

We did not find any relationship between the FSH levels and thiol levels in light of the study conducted by Işık *et al.* [23]. Moreover, disulfide levels in the low ovarian reserve group were not different from that in other patients.

One of the most significant findings of our study was that no relationship exists between the pregnancy results and disulfide level. In addition, there is a correlation between disulfide and M2 oocytes. This result could show that the oxygen radicals have a role in oocyte maturation, and low levels of OS are beneficial.

However, the S–S values are higher in the groups with high M2 values (unexplained & male factor) than in the DOR group, with its low S–S and low M2 values. However, statistical significance between the groups was not observed for S–S levels.

The limitation of our study is that the perinatal results are not mentioned, and we did not measure disulfide levels in follicular fluid or endometrial fluids. This warrants further study.

## Conclusion

The results of study are interesting because there were no relationship between pregnancy results but there was a correlation between disulfide and M2 oocyte. However, the belief that ‘the ratio of S–S increases, the ratio of M2 also increases’ can not be justified based on the correlation between them. Therefore, further studies with larger sample size would be useful to further elucidate the roles of disulfide levels in oocyte maturation.

## Future perspective

OS is an important problem in human reproduction. There are a lot of studies concerning it in the literature. Thiol/disulfide homeostasis is a cheaper way of showing OS. Further studies are needed to understand and elucidate the effects of OS, using this method. It is hoped that new therapies will be found to enhance IVF success, by resolving the OS problem.

**Summary points**

- Oxidative stress (OS) is an important issue in success of *in vitro* fertilization.
- There are a lot of methods to measure the OS.
- Thiol/disulfide homeostasis is one of the cheaper methods of measuring OS.
- In this study we included 62 *in vitro* fertilization-intracytoplasmic sperm injection patients.
- Serum samples were taken from the patients on the day of oocyte pick-up.
- Serum thiol/disulfide homeostasis parameters were measured.
- There was no relationship between pregnancy results, but there was a correlation between disulfide and M2 oocytes.
- According to our results OS be beneficial for oocyte maturation and M2 oocytes.
